# Heparin Increases HLA-G Levels in Primary Antiphospholipid Syndrome

**DOI:** 10.1155/2012/232390

**Published:** 2012-05-13

**Authors:** Jozélio Freire de Carvalho, Ricardo M. de Oliveira, Carlos Ewerton Maia Rodrigues, Andréa Glezer, Eloísa Bonfá, Rosa Maria Rodrigues Pereira

**Affiliations:** ^1^Reumatologia Divisão, Faculdade de Medicina, Universidade de São Paulo, Sala 3105, Avenida Dr. Arnaldo, 455, 01246-903 São Paulo, SP, Brazil; ^2^Laboratório Clínico, RDO Diagnósticos, Avenida Brasil 1150, 01430-001 São Paulo, SP, Brazil; ^3^Edocrinologia Divisão, Hospital das Clinicas da Faculdade de Medicina, Universidade de São Paulo, Avenida Dr Enéas de Carvalho Aguiar 44, 05403-000 São Paulo, SP, Brazil

## Abstract

*Objectives*. The aim of this study was to investigate the HLA-G serum levels in Primary Antiphospholipid Syndrome (PAPS) patients, its impact on clinical and laboratory findings, and heparin treatment. *Methods*. Forty-four PAPS patients were age and gender matched with 43 controls. HLA-G serum levels were measured using an enzyme-linked immunosorbent assay (ELISA). *Results*. An increase in soluble HLA-G levels was found in patients compared to controls (3.35 (0–22.9) versus 1.1 (0–14), *P* = 0.017). There were no significant differences in HLA-G levels between patients with and without obstetric events, arterial thrombosis, venous thrombosis, or stroke. Sixty-six percent of patients were being treated with heparin. Interestingly, patients treated with heparin had higher HLA-G levels than ones who were not treated with this medication (5 (0–22.9) versus 1.8 (0–16) ng/mL, *P* = 0.038). Furthermore, patients on heparin who experienced obstetric events had a trend to increased HLA-G levels compared to patients who were not on heparin and did not have obstetric events (5.8 (0–22.9) versus 2 (0–15.2) ng/mL, *P* = 0.05). *Conclusion*. This is the first study to demonstrate that serum HLA-G levels are increased in APS patients. We also demonstrated that heparin increases HLA-G levels and may increase tolerance towards autoantigens.

## 1. Introduction

 Human leukocyte antigen- (HLA-) G is a nonclassical HLA class Ib molecule from the major histocompatibility complex. It was originally discovered in the context of protecting a fetus from the mother's immune system [1]. Indeed, HLA-G seems to be involved in the induction and maintenance of tolerance between the mother's immune system and the semiallogeneic fetus at the fetal-placental interface; it also seems to play an important role in embryo implantation [2]. Beyond its role in fetal-maternal tolerance, HLA-G exerts tolerogenic functions in transplant acceptance and in tumoral and viral immune escape [1]. HLA-G antigens can affect the cytotoxicity of natural killer and CD8^+^ T cells, CD4^+^ T-lymphocyte functions, and dendritic cell maturation [3].

 Recent studies have demonstrated the altered expression of HLA-G in autoimmune diseases, such as multiple sclerosis, rheumatoid arthritis, and systemic lupus erythematosus (SLE), suggesting a role for this molecule in the pathophysiology of autoimmune diseases [4–7]. Antiphospholipid syndrome (APS) is an autoimmune disease frequently associated with obstetric complications including fetal loss, recurrent spontaneous abortions, and placental insufficiency. Consequently, alterations in the HLA-G system might be a possible mechanism involved in the etiology of this autoimmune disorder. However, there is no other study that evaluated HLA-G levels in APS patients. This report represents the first investigation of the role of HLA-G in APS.

 The present study was therefore undertaken to determine the serum HLA-G levels in patients with PAPS and to understand the possible associations between HLA-G levels and clinical and laboratory findings in PAPS.

## 2. Methods

### 2.1. Patients

This is a cross-sectional study that included 44 patients with PAPS, according to Sapporo criteria [8], and 43 age- and race-matched healthy subjects who served as controls. All patients were followed at the Antiphospholipid Syndrome Outpatient Clinic within the Division of Rheumatology at the Hospital das Clínicas da Faculdade de Medicina da Universidade de São Paulo.

Patients who had secondary causes of APS, such as SLE, rheumatoid arthritis (RA), or vasculitis, were excluded from this study.

All subjects provided informed consent, and the study was approved by the Ethics Committee of the University Hospital.

### 2.2. Data Collection

Demographic data for patients and controls were obtained through interviews and physical examinations. Variables collected included age in years, gender, race, and weight. Clinical and therapeutic data, including disease duration, thrombotic and obstetric manifestations, and medication history including administration of warfarin, heparin, aspirin, anticonvulsants, and antimalarials, were obtained from the medical records and interviews.

### 2.3. Measurement of sHLA-G

Capture enzyme-linked immunosorbent assay (ELISA) was used to measure HLA-G levels. Briefly, 96-well microplates (Nunc-C8 MaxiSorp BreakApart Immunomodule, Roskilde, Denmark) were coated for 72 h with 100 *μ*L/well of monoclonal antibody MEM-G/9 (MCA2004-Serotec, Raleigh-NC, USA) in 10 *μ*g/mL BBS (pH 8.4) and incubated at 4°C. After three washes with PBS, 200 *μ*L of a solution containing 5% bovine serum and 10% sucrose in PBS was added to the wells, and the plates were incubated for 12 h at 4°C. Before use, this solution was aspirated, and plates were incubated for 2 h at 37°C. Fifty *μ*L of calibration solution (diluted to 0, 3.125, 6.25, 12.5, and 25.0 ng/mL) from JEG-3 cells (human placental choriocarcinoma), or sera from patients and controls (undiluted), was added in duplicate and followed by an incubation for 2 h at room temperature (RT). After three washings with PBS, 50 *μ*L of a peroxidase-conjugated IgG fraction of rabbit anti-beta-2-microglobulin (Rockland Inc., Gilbertsville, PA, US), diluted 1 : 500 in PBS with 10% normal rabbit serum, was added and incubated for 1 h at RT. The reaction was developed with 100 *μ*L of TMB, and after 30 min the reaction was stopped with H_2_SO_4_ 1 N. Plates were read at an optical density (OD) of 450 nm (EL Universal Microplate Reader, BioTek Instruments Inc., Winooski, VT, USA). HLA-G levels were calculated using a standard curve and results were expressed in ng/mL [9, 10]. The intra-assay CV was 3.2 to 6.9% and the interassay 7.7 to 13.9%.

### 2.4. Antiphospholipid Antibodies

 IgG and IgM anticardiolipin antibodies (aCL) were tested at least twice, with an interval of 12 weeks between measurements, using an ELISA as previously described [11]. According to the manufacturer's recommendations, only titers of aCL > 20 U/mL were considered positive. Lupus anticoagulant was measured according to international guidelines using activated partial thromboplastin time (APTT, Diagnostica Stago, France) and diluted Russell's viper venom time (dRVVT, Trinity Biotech, Wicklow, Ireland) [12].

### 2.5. Statistical Analysis

Data are reported as a median (minimum-maximum) for HLA-G serum levels, mean (± standard deviation), or a percent. Variables were compared between patients and controls using Student's *t*-test or the Chi-square test. *P* values < 0.05 were considered significant.

## 3. Results

Patients and controls were similar in terms of age (42.5 ± 12.0 versus 39.8 ± 11.2 years, *P* = 0.29, *t-*test), frequency of female gender (86.4 versus 78.9%, *P* = 0.47, Chi-square test), and Caucasian race (90.9 versus 84.2%, *P* = 0.42, Chi-square test). Mean disease duration was 97.9 ± 70.3 months.

Arterial thrombosis was observed in 59.1% of patients, venous thrombosis in 59.1%, obstetric events in 38.6%, thrombocytopenia in 18.2%, *livedo reticularis* in 34.1%, stroke in 43.2%, Sneddon's syndrome in 24.5%, pulmonary thromboembolism in 27.3%, deep venous thrombosis in 54.5%, and angina in 9.1%. In patients with PAPS, 38.6% reported prior smoking history and 11.4% were current smokers (Table 1). Lupus anticoagulant was positive in 60.5% of patients, IgG anticardiolipin in 36.8%, and IgM anticardiolipin antibodies in 10.5%. Sixty-six percent of patients were being treated with heparin, and 40.9% were being treated with chloroquine diphosphate.

**Figure 1:**
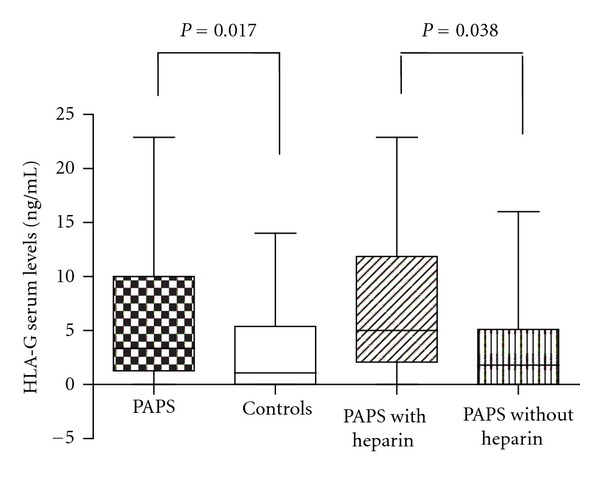
HLA-G serum levels in 44 PAPS patients and 43 healthy controls, and in PAPS patients with and without heparin.

Notably, significantly increased levels of soluble HLA-G were noted in PAPS patients compared to controls (3.35 (0–22.9) versus 1.1 (0–14), *P* = 0.017, Mann-Whitney test) (Table 1, Figure 1). Serum HLA-G levels were similar in patients with and without obstetric events, arterial thrombosis, venous events, and stroke (*P* > 0.05) (Mann-Whitney test) (Table 2).

Interestingly, patients taking heparin had higher HLA-G levels than subjects who were not using this medication (5 (0–22.9) versus 1.8 (0–16) ng/mL, *P* = 0.038, Mann-Whitney test) (Figure 1). Furthermore, patients taking heparin who had obstetric events (*n* = 13) had a trend to increased levels of HLA-G compared to other patients (*n* = 25) (5.8 (0–22.9) versus 2 (0–15.2) ng/mL, *P* = 0.05, Mann-Whitney test).

Patients taking heparin who experienced arterial thrombosis (*n* = 16) had similar levels of HLA-G compared to the other patients (*n* = 28) (4.55 (0–15.2) versus 3.2 (0–22.9) ng/mL, *P* = 0.557, Mann-Whitney test), and patients taking heparin who experienced venous thrombosis (*n* = 18) had comparable levels of HLA-G compared to the others (*n* = 26) (3.6 (0–22.9) versus 3.35 (0–15.9) ng/mL, *P* = 0.990, Mann-Whitney test). 

## 4. Discussion

This is the first study to demonstrate higher levels of HLA-G in PAPS patients compared to healthy subjects.

One advantage of this study was the inclusion of exclusively primary APS patients because it is clear that SLE can alter HLA-G levels [6, 7]. Indeed, previous studies of HLA-G in SLE have presented conflicting results [6, 7, 13]. Lower plasma levels of soluble HLA-G were more frequent in patients with SLE [6]. Wu et al. [13] demonstrated a significant increase in the expression of soluble HLA-G in patients with SLE; high levels of soluble HLA-G were also associated with more active disease and more neurologic involvement. It is possible that enhanced expression of soluble HLA-G in SLE contributes to a mechanism that restores tolerance towards autoantigens and counteracts inflammation. However, the participation of this molecule in the pathologic process of SLE has not been excluded. Thus, environmental and/or ethnicity-specific factors may be contributing to such conflicting results.

Recent studies have demonstrated that HLA-G is induced with the development of inflammatory pathologies such as myositic lesions, psoriatic lesions on skin, and multiple sclerosis [4, 14, 15]. Furthermore, HLA-G expression on the surface of epithelial intestinal cells seems to play a role in the suppression of proinflammatory cytokines in ulcerative colitis [16]. It has been suggested that HLA-G expression plays a role in a possible mechanism of tissue protection against autoimmune inflammatory responses, therefore acting as a mechanism of immune surveillance [17, 18]. In this regard, a combination of genes, instead of a single gene, predisposes one to an immunologic disorder that leads to defective mechanisms of immunological tolerance, allowing antibody production against autoantigens, immune complex formation, and deposition [19].

Our study provided an opportunity to assess the possible connections between PAPS and HLA-G levels. While we demonstrated that HLA-G levels are increased in PAPS patients, we did not observe a difference in the HLA-G levels in patients who had clinical manifestations of PAPS, including obstetric events.

 Recent evidence suggests that HLA-G may be involved in the suppression of immune responses including inhibition of the proliferation of CD4^+^ T cells, induction of CD4^+^ T-cell anergy, and differentiation of CD4^+^ T cells into suppressive cells [20]. A similar process was suggested to explain the higher soluble HLA-G expression in correlation with RA disease activity [21].

One possible explanation is that heparin could increase HLA-G expression on target tissues. In fact, Baczyk et al. demonstrated that heparin plus fibroblast growth factor 4 (FGF4) induced an increase of HLA-G expression with more invasiveness of trophoblast tissue [22].

The most interesting finding in this study was that patients taking heparin had higher HLA-G than patients who were not treated with this medication. Furthermore, patients on heparin who experienced obstetric events had a trend to increased levels of HLA-G compared to patients not taking heparin who did not have obstetric events. These findings suggest a possible enhanced expression of soluble HLA-G in PAPS as part of a mechanism to restore the tolerance process towards autoantigens; it also demonstrates that heparin increases HLA-G levels and may increase this immunologic aberrancy. Kovats et al. [2] demonstrated that HLA-G is expressed in cytotrophoblasts. They identified a molecule and a gene that could be involved in placental-maternal interaction and could be studied quantitatively and qualitatively in connection with certain types of unexplained infertility and spontaneous abortion [23].

 In summary, this study demonstrated higher levels of HLA-G in PAPS, particularly in patients with obstetric events. We also demonstrated that heparin increases HLA-G levels and may increase tolerance towards autoantigens.

## 5. Rheumatology Key Messages

- Serum HLA-G levels are increased in antiphospholipid syndrome patients in comparison to healthy controls.
- Heparin treatment increases HLA-G levels in antiphospholipid syndrome patients with obstetric events.

## Figures and Tables

**Table 1:** Demographic, anthropometric, clinical, and laboratory features of the 44 PAPS patients and 43 healthy controls that provided samples for the measurement of HLA-G levels.

	PAPS *n* = 44	Controls *n* = 43	*P*
Age, years	42.47 (12.0)	39.83 (11.2)	0.29
White race, %	90.9	84.2	0.42
Arterial events, %	59.1	NA	—
Venous events, %	59.1	NA	—
Obstetric events, %	38.6	NA	—
Thrombocytopenia, %	18.2	NA	—
Livedo reticularis, %	34.1	NA	
Stroke, %	43.2	NA	—
Sneddon's syndrome, %	24.5	NA	—
Pulmonary thromboembolism, %	27.3	NA	—
Deep venous thrombosis, %	54.5	NA	—
Angina, %	9.1	NA	
Disease duration, months	97.9 (70.3)	NA	—
Previous history of smoking, %	38.6	NA	—
Current smoking, %	11.4	NA	—
Heparin use, %	66	NA	—
Current chloroquine use, %	40.9	NA	—
HLA-G, ng/mL^#^	3.35 (0–22.9)	1.1 (0–14)	0.017

NA: not applicable.

Data expressed as a mean (standard deviation), ^#^median (minimum-maximum), or a percentage.

**Table 2:** Comparison of HLA-G levels between primary antiphospholipid syndrome patients (PAPS) with and without clinical events.

	HLA-G levels in PAPS with clinical events	HLA-G levels in PAPS without clinical events	*P*
Obstetric event	5.4 (0–22.9)	2.0 (0–15.2)	0.206
Arterial event	2.5 (0–15.2)	4.8 (0–22.9)	0.304
Venous event	2.9 (0–22.9)	4.15 (0–15.2)	0.574
Stroke	2.4 (0–16)	5 (0–22.9)	0.18

Data expressed as a median (minimum-maximum) in ng/mL.
